# Sourcing Interchangeability in Commercial Chitosan: Focus on the Physical–Chemical Properties of Six Different Products and Their Impact on the Release of Antibacterial Agents

**DOI:** 10.3390/polym17070884

**Published:** 2025-03-26

**Authors:** Isabela Tavares Rampim, Helton José Wiggers, Cecilia Zorzi Bueno, Pascale Chevallier, Francesco Copes, Diego Mantovani

**Affiliations:** 1 Laboratory for Biomaterials and Bioengineering (LBB-BPK), Associação de Ensino, Pesquisa e Extensão BIOPARK, Max Planck Avenue, 3797, Building Charles Darwin, Toledo 85919-899, PR, Brazil; isabela.rampim@outlook.com (I.T.R.); cecilia.bueno@bpkedu.com.br (C.Z.B.); 2 Laboratory for Biomaterials and Bioengineering (LBB-UL), Department of Min-Met-Materials Engineering & CHU de Quebec Research Center, Division Regenerative Medicine, Laval University, Quebec City, QC G1V0A6, Canada; pascale.chevallier@crchudequebec.ulaval.ca (P.C.); francesco.copes.1@ulaval.ca (F.C.)

**Keywords:** natural polymers, chitosan, interchangeability, drug release systems

## Abstract

Sourcing and batch differences are often cited as intrinsic drawbacks for all natural polymers. Chitosan makes no exception. Chitosan is a biocompatible and biodegradable biopolymer with high potential for several biomedical applications, especially for releasing drugs and bactericidal and virucidal agents. Despite the potential of chitosan as a matrix for producing antibacterial films, the variability in its composition, stemming from its natural sources, can hinder the translation from bench to industry. To overcome this concern, we conducted a study to access the interchangeability of chitosan for the development of antibacterial drug release systems, in particular one system crosslinked with tannic acid and iron sulfate. Chitosans from different suppliers were characterized and used to synthetize films containing gentamicin, according to a previously reported protocol. The impact of molecular weight (M_W_), deacetylation degree and purity on film properties and antibiotic release kinetics was assessed and results were compared. The films exhibited different initial bursts followed by similar sustained release profiles. All films exhibited antibacterial activity against both *E. coli* and *S. aureus* for at least 42 days. Moreover, films were cyto- and hemocompatible. Therefore, despite some differences in physicochemical properties, the interchangeability among the studied chitosan suppliers to produce antibacterial films is feasible, and the final product properties and performances are not significantly altered.

## 1. Introduction

Chitosan is a polycationic copolymer composed of N-acetyl-D-glucosamine and D-glucosamine units available in different proportions depending on the degree of acetylated moieties [1]. This natural polymer is obtained by the deacetylation of chitin, the second most abundant polysaccharide in nature behind cellulose. Chitin can be found in the exoskeleton of crustacea, insect cuticles, algae and fungi cell walls. Crab shells, shrimp shells, fungi and mushrooms are the most common sources of chitin [2,3].

As a unique and versatile compound, chitosan, alone or in composites, is used in various fields, and its modification/functionalization gives it new properties. In particular, chitosan is widely studied in the pharmaceutical and biomedical fields due to its biological properties such as biocompatibility, i.e., non-toxicity, low immunogenicity and allergenicity, recognized antibacterial properties, biodegradability and facility of processing [4,5,6,7]. Its use in multiple fields such as drug delivery [8,9,10,11], vaccines [12,13], wound dressings [14,15,16], gene delivery [17,18,19], scaffolds for tissue regeneration [20,21,22,23], tissue engineering, sensors and implant design [24,25] is, however, often restricted on an industrial scale due to its high variability, caused by chitin extraction sources [5], but also due to its highly hygroscopic nature. In addition, chitosan’s physicochemical properties such as molecular weight, polydispersity, degree of deacetylation and purity levels vary considerably from batch to batch [26]. This complicates the task of standardizing finished biomedical products and consistently guaranteeing the quality. This is worsened by the risk of supply chain disruption, which can eventually lead to supplier change and, consequently, to a modification of chitosan’s properties, causing the above-mentioned alterations and affecting product performance.

In this perspective, a practical challenge for industries is to select chitosan suppliers for their biomedical products. However, chitosan manufacturers generally provide incomplete data on product specifications, which can be misleading when it comes to supplier selection and product validation [27]. Marques et al. reviewed how the lack of chitosan characterization hampers the safe-by-design approach, a guideline aimed at helping industries to identify risks and uncertainties early in the research and development phase [28]. Moreover, Bellish et al. highlighted the importance of an accurate correlation between chitosan’s chemical structure and the effective biological responses of finished chitosan-based biomedical products [5]. Thus, an understanding of the physicochemical variability of chitosan from different suppliers in the early stages of R&D can potentially increase the chances of processes translation in the pharmaceutical and biomedical industries.

Although there is some research comparing the effects of different molecular weight (M_W_) and degree of deacetylation (DDA) of chitosan on different products, such as hydrogels, films for drug release and food packaging [29,30,31,32], studies comparing chitosan from different suppliers are still rare. Therefore, based on a previously reported protocol to prepare crosslinked chitosan films loaded with an antibiotic [8], the research question in this study was as follows: is it possible to obtain chitosan films with similar physicochemical and antibacterial properties using chitosans with different characteristics from different suppliers?

The selection of the different chitosans was based on the information given by their suppliers. Usually, chitosans’ certificates of analysis provide only the degree of deacetylation and the viscosity of a 1% chitosan solution at 25 °C dissolved in 1% acetic acid. Molecular weight is rarely reported as a numeric value and sometimes refers to a range of molecular weights (low, medium or high). However, chitosan classification according to its molecular weight range is not well defined in the literature, and the available data are relative and sometimes even confusing [33].

In this sense, the authors chose to adopt chitosan classification according to Sigma-Aldrich, the supplier of the standard polymer currently used by the research group [8,34]. According to Sigma-Aldrich, there are three main viscosity ranges, which are directly related to molecular weight: low viscosity (20–300 cP) with molecular weight 50–190 kDa, medium viscosity (200–800 cP) with molecular weight 190–310 kDa and high viscosity (800–2000 cP) with molecular weight 310–375 kDa [35]. In this work, six different chitosans with viscosities ranging from 36 cP to 1100 cP were chosen, encompassing from low to high viscosity and, therefore, from low to high molecular weight.

The selected chitosans were characterized regarding their molecular weight, degree of deacetylation, moisture level and impurities, following the recommendations of ASTM [36]. Specifically, the impurity levels were quantified by measuring the contents of ash, chloride, protein and metals, which can be present as residues of the chitosan production process [23,37] and should therefore be minimized to avoid undesirable tissue reactions considering biomedical applications. The interchangeability between the chitosan suppliers was verified by producing chitosan films crosslinked with tannic acid and iron and loaded with the antibiotic gentamicin [8] and characterizing them in terms of their physicochemical and biological properties.

## 2. Materials and Methods

### 2.1. Materials 

Six different chitosans (CS), from low to high viscosity, were used in this study, and their given acronyms are shown in Table 1. The selected chitosans were of medium molecular weight from Sigma-Aldrich, Shanghai, China; Chytolytic, Toronto, Canada; Zhejiang Golden Shell Pharmaceutical, Yuhuan, China; Fingres, Wuxi, China; Quitomax, Palotina, Brazil; and Jiangsu Chitin Biotech, Yancheng, China.

Gentamicin sulfate (GS) (Sigma-Aldrich, St. Louis, MO, USA), acetic acid 99.7% (Synth, Diadema, Brazil), tannic acid (TA) (ACS reagent, Sigma-Aldrich, Wuxi, China), iron sulfate heptahydrate 99.0% (Fe) (Êxodo Científica, Sumaré, Brazil), phosphate-buffered saline (PBS) (Sigma, Gillingham, UK), acetonitrile >99.9% (Merck, Darmstadt, Germany), ethanol (Synth, Diadema, Brazil), formic acid ≥95% (Synth, Diadema, Brazil), trifluoroacetic acid ≥99.5% (Sharlau, Barcelona, Spain), Mueller-Hinton broth (Difco, Sparks, NV, USA), Mueller-Hinton agar (Kasvi, Madrid, Spain), *Staphylococcus aureus* ATCC 6538 and *Escherichia coli* ATCC 8739 (Lab-Elite™, St Cloud, MN, USA), glycerol ≥99.8% (Neon, Suzano, Brazil), Dulbecco’s modified Eagle’s medium (DMEM) (Gibco, Invitrogen Corporation, Burlington, ON, Canada), resazurin sodium salt (Sigma-Aldrich, Oakville, ON, Canada), human dermal fibroblasts C0045C (Gibco, Invitrogen, Burlington, ON, Canada), fetal bovine serum (FBS) (Gibco, Invitrogen Corporation, Burlington, ON, Canada), trypsin (Gibco, Invitrogen Corporation, Burlington, ON, Canada), penicillin (Gibco, Invitrogen Corporation, Burlington, ON, Canada) and streptomycin (Gibco, Invitrogen Corporation, Burlington, ON, Canada) were used in this work. All chemicals and reagents were used as received.

### 2.2. Chitosan Characterization 

#### 2.2.1. Molecular Weight

Chitosan’s molecular weights were evaluated by size-exclusion chromatography (SEC) (e2695, Waters, Milford, CT, USA) connected to a multiangle laser light-scattering detector (MALS) (miniDawn, RID Optilab, Wyatt Technology, Santa Barbara, CA, USA) [38]. Samples were eluted through a pre-column (LB-G 6B) and two separation columns in sequence (Shodex OHPAK LB-803 and LB-806M, Resonac, New York, NY, USA). The mobile phase was acetate buffer (acetic acid 0.3 M, sodium acetate 0.2 M, pH 4.5, filtered at 0.2 µm) at a flow rate of 0.5 mL/min. Chitosan solutions at 4 mg/mL were prepared in acetate buffer and filtered in sintered glass before analysis. The volume of the injected samples was 18 µL, and the analysis was conducted at 35 °C for 60 min for each run. The detection wavelength was 658.8 nm. All measurements were performed in triplicates.

#### 2.2.2. Deacetylation Degree

The deacetylation degree was determined by potentiometric titration [39]. Briefly, a chitosan solution of 4 mg/mL in 0.1 M HCl was titrated with 0.1 M NaOH. A titration curve of pH vs. NaOH titration volume was generated. The curve resulted in two inflection points, where the first one represents the titration of excess HCl and the second represents the titration of the chitosan deacetylated units. The difference between these volumes is used to calculate the deacetylation degree following Equation (1):

(1) $$DDA\left(\%\right)=\left(\frac{\Delta V_{NaOH}\times M_{NaOH}\times 161}{m_{C}}\right)\times 100$$

where $\Delta V_{NaOH}$ is the difference between the two inflection points, $M_{NaOH}$ is the NaOH molarity, 161 is the chitosan deacetylated monomer molecular mass and $m_{C}$ is the mass of the sample. Therefore, the dividend of this equation is the total mass of D-glucosamine units present in the sample, and the divisor is the total mass of the sample, which encompasses both deacetylated (D-glucosamine) and acetylated (N-acetyl-D-glucosamine) units. The quotient result is the degree of deacetylation. The experiments were performed in triplicates.

#### 2.2.3. Moisture

To determine moisture, 1 g of chitosan was dried at 105 °C until achieving constant weight, and the final moisture content was determined according to Equation (2):

(2) $$\%Moisture=\frac{w_{0}\;{-w}_{d}}{w_{0}}\times 100\%$$

where $w_{d}$ is the dried weight and $w_{0}$ is the initial, or wet, weight. Experiments were carried out in triplicates [40].

#### 2.2.4. Ash

The ash content was assessed by weighing 1 g of chitosan in a crucible that was previously heated and cooled to room temperature. Then, the samples were heated in a muffle at 600 °C for 6 h. The samples were cooled to room temperature in desiccators and weighted [41]. The ash content was calculated according to Equation (3):

(3) $$\%Ash=\frac{w_{r}}{w_{0}}\times 100\%$$

where $w_{r}$ is the residue weight and $w_{0}$ is the initial weight. The experiments were carried out in triplicates.

#### 2.2.5. Chloride 

Chloride content was determined by the Mohr argentometric method as follows: 0.5 g of chitosan was weighed in a crucible, heated in a muffle at 600 °C for 6 h in and cooled in desiccator until room temperature. Then, 50 mL of warm water was added to the ash, followed by the addition of 4 drops of K_2_CrO_4_ as the indicator and titration with 0.1 M AgNO_3_. The chloride content was calculated following Equation (4):

(4) $$\%Cl=\frac{V\times f\times 0.584}{w_{0}}$$

where V is the volume of titration, f is the correction factor of the AgNO_3_ and $w_{0}$ is the initial weight. The test was carried out in triplicates.

#### 2.2.6. Protein 

Protein content was measured by elemental analysis [42] using a Vario MACROCube (Elementar, Hanau, Germany) with detection limit <50 ppm. Samples were previously conditioned in tin capsules and weighed (~70 mg). After, the samples were pressed and taken to the equipment for analysis. The temperatures used were 1150 °C for the combustion tube, 850 °C for the reduction tube, 240 °C for the CO_2_ column, 150 °C for the H_2_O column and 230 °C for the SO_2_ column. The experiments were performed in duplicates.

Protein content (%*P*) was calculated according to Equation (5).

(5) $$\%P=\left(\%N-N_{T}\right)\times 6.25$$

where %*N* is the total nitrogen percentage measured by elemental analysis. The number 6.25 refers to the theoretical nitrogen percentage in proteins [42].

*N_T_* is the theoretical nitrogen percentage calculated based on chitosan’s degree of deacetylation (*DDA*) and the nitrogen percentage in total acetylated chitosan (*N_chitin_* = 6.89%) and total deacetylated chitosan (*N_chitosan_* = 8.69%). The calculation was performed according to Equation (6) [43].

(6) $$N_{T}=\left(N_{chitosan}-N_{chitin}\right)\times DDA+N_{chitin}$$

#### 2.2.7. Metals

Heavy metals were assessed in an inductively coupled plasma–optical emission spectrometer (ICP-OES) (PerkinElmer, Optima 8000, Waltham, MA, USA). The samples were prepared using 0.5 g of chitosan with 3 mL of HNO_3_ and 2 mL of ultrapure water. Then, solutions were decomposed by microwave radiation and digested at 200 °C. This solution was then washed with ultrapure water. Arsenic (As, emission wavelength (ew) = 188.979 nm) and mercury (Hg, ew = 253.652 nm) were analyzed using chemical vapor generation (CVG). Cadmium (Cd, ew = 228.802), cobalt (Co, ew = 238.892 nm), chromium (Cr, ew = 267.16 nm), copper (Cu, ew = 324.752 nm), nickel (Ni, ew = 231.604 nm), lead (Pb, ew = 220.353 nm) and tin (Sn, ew = 235.485 nm) were analyzed after nebulization. The limits of detection (LoD) and quantification (LoQ) were determined based on the calibration curve using Equations (7) and (8).

(7) $$LoD=\frac{3.3\times sd}{Slope}\;$$

(8) $$LoQ=\frac{10\times sd}{Slope}\;$$

where sd is the standard deviation of the smaller concentration from the calibration curve.

### 2.3. Film Preparation

Chitosan films were synthetized according to a previously developed protocol [8]. Herein, the film composition contains a tannic acid–iron sulfate complex as a crosslinker and is loaded with GS. The preparation of the stock solutions was as follows: CS 15 mg/mL in acetic acid 1% *v*/*v*, Fe 3 mg/mL, TA 50 mg/mL and GS 10 mg/mL in ultrapure water. Next, 6.67 mL of CS solution was mixed in a beaker, with the subsequent addition of 0.4 mL of TA, 1.67 mL of Fe and 1.0 mL of GS under magnetic stirring. Ultrapure water was added to adjust the final volume to 10 mL. The samples were deposited into 90 mm Petri dishes and dried at 37 °C until a constant mass was reached.

### 2.4. Film Characterization 

#### 2.4.1. Thickness 

Thickness was assessed with a digital micrometer (Mitutoyo, Kawasaki, Japan) in five different points and reported as the average and standard deviation [8].

#### 2.4.2. Swelling and Mass Loss in PBS 

To study swelling and mass loss, the films were cut in small 1 mg squared samples and submerged in 2 mL of PBS 1× at 37 °C and 150 rpm for 24 h. For swelling measurements, the samples were then washed twice with distilled water and blotted with filter paper to remove excess water. Finally, the swollen samples were weighed, and their swelling ratio was calculated using Equation (9):

(9) $$SR=\frac{w_{s}-w_{0}}{w_{0}}\times 100\%$$

where $w_{0}$ is the sample’s initial weight and $w_{s}$ is the sample’s weight after swelling [8].

For mass loss measurements, the samples were washed twice with distilled water and dried at 37 °C until achieving constant mass. Mass loss was determined according to Equation (10):

(10) $$ML=\frac{w_{0}-w_{d}}{w_{0}}\times 100\%$$

where $w_{0}$ is the initial weight and $w_{d}$ is the final weight. Measurements were performed in quintuplicates [8].

#### 2.4.3. Contact Angle 

Static contact angle was assessed in a VCA 2500 XE system (AST^®^, Billeria, MA, USA) at room temperature. Ultrapure water droplets of 1 μL were deposited onto three distinct regions of each sample. The experiments were performed in triplicates [8].

### 2.5. Antibiotic Release in PBS

To study the antibiotic release kinetics, small chitosan films were prepared inside glass vials, resulting in a surface area of 1.77 cm^2^. Then, each vial was filled with 2 mL of PBS 1×, and the systems were kept in an incubator at 150 rpm and 37 °C. Release media were collected at specific intervals, i.e., immediately (0 h) and after 1 h, 4 h, 6 h, 1 day, 3 days, 7 days and then every 7 days. After each collection, 2 mL of fresh PBS solution was added to the samples. The collected samples were stored at −20 °C and thawed before quantification. Experiments were carried out in triplicates.

GS quantification was performed using high-performance liquid chromatography (HPLC 2696, Waters) coupled to mass spectrometry (Micromass Quattro Micro API) (Waters, Milford, CT, USA). Water–acetonitrile (20:80 *v*/*v*), 0.5% (*v*/*v*) trifluoroacetic acid and 0.5% (*v*/*v*) formic acid were used as the mobile phase at a constant flow rate of 0.5 mL/min. The separation column was a Triart C18 250 mm × 4.6 mm, 3 μm (YMC, Kyoto, Japan), kept at 30 °C. Mass spectrometry conditions were as follows: nitrogen as drying gas at 400 L/h and 350 °C, vaporizer temperature of 150 °C, capillary voltage of 3000 V, multiple reaction monitoring dwell time of 600 ms, fragmentation voltage of 50 V, collision energy of 10 V and ESI positive mode. The injection volume was 20 μL. The quantification and detection limits were 0.105 and 0.031 μg/mL, respectively [8].

### 2.6. Antibacterial Activity

#### 2.6.1. Bacteria Stock Preparation 

*Staphylococcus aureus* (ATCC 6538) and *Escherichia coli* (ATCC 8937) were cultured on 90 mm Petri dishes containing fresh sterile Mueller-Hinton agar. The dishes were incubated in an inverted position overnight at 37 °C. A single colony was transferred to 20 mL of fresh sterile Mueller-Hinton broth and incubated overnight at 37 °C and 150 rpm. Then, 15% *v*/*v* sterile glycerol was added for cryoprotection, and aliquots of the bacterial suspension were frozen at −20 °C. The number of CFU/mL of the bacteria stocks were determined using the log dilution method. Likewise, the bacteria were prepared prior to assay inoculation.

#### 2.6.2. Indirect Antibacterial Activity over Time 

For antibiotic release in culture media, films were prepared in sterilized glass vials. Then, 2 mL of sterile Mueller-Hinton broth was added to each vial, incubated at 180 rpm and 35 °C. The eluates were collected at different time points (6 h, 1 day, 3 days, 7 days, 14 days, 21 days and every 7 days until antibacterial activity was not observed) and replaced with 2 mL of fresh culture media. All the eluates were stored at −20 °C before testing.

The bacterial stocks were thawed and diluted in sterile Mueller-Hinton broth to c.a 1 × 10^6^ CFU/mL (OD_600_ c.a. 0.020). Subsequently, 100 µL of the collected eluates was mixed with 100 µL of inoculum in a 96-well plate. Negative controls (blanks) were prepared by adding 100 µL sterile antibiotic-free Mueller-Hinton broth to the inoculum. Positive controls were prepared by adding gentamicin at 10 µg/mL. Plates were incubated at 35 °C at 200 rpm until obtaining OD_600_ 0.6–0.8. After incubation, the OD_600_ for all the samples was determined and compared against the negative controls. Then, the bacterial survival was determined using Equation (11). The samples were measured out in triplicates [8].

(11) $$\%Bacterial\;survival=\frac{{film\;sample\;OD}_{600}}{{blank\;OD}_{600}}\cdot 100\%$$

### 2.7. Biocompatibility

Before the cytotoxicity and hemocompatibility test, all samples were sterilized on both sides with UV-C irradiation at 254 nm. Each side underwent 2 cycles of 15 min UV irradiation. Afterwards, samples were stored in sterile 24 multi-well plates until analysis.

#### 2.7.1. Cell Culture 

Human dermal fibroblasts (HDFs) were grown in Dulbecco’s modified Eagle’s medium (D-MEM) enriched with 10% fetal bovine serum (FBS), penicillin (100 U/mL) and streptomycin (100 U/mL). Cells were maintained at 37 °C in a saturated atmosphere at 5% CO_2_. The culture medium was replaced every 48 h, and cells were allowed to proliferate until reaching 85–90% of confluence. Finally, cells were enzymatically detached from the plates (0.05% trypsin). Samples were either re-plated at a ratio of 1:3 or used for experiments. The cells used for the experiments were from passage 7.

#### 2.7.2. Indirect Cytotoxicity Assay 

The indirect cytotoxicity assay was conducted following the ISO guidelines [44]. Briefly, 1 cm^2^ samples (*n* = 3) were submerged in 660 µL of D-MEM supplemented with 1% penicillin–streptomycin (P/S) and incubated in a saturated atmosphere at 5% CO_2_ at 37 °C for 1 day. After incubation, the media from each sample were collected for the cytotoxicity test. Before analysis, the extracted media were supplemented with 10% FBS. HDFs were seeded at 20,000 cells/cm^2^ in a 96-well microplate containing 100 µL of complete medium per well. The plate was incubated at 37 °C and 5 % CO_2_ for 24 h. Later, the medium was discarded, and 100 µL of the extracts was added to the wells containing the cells, followed by incubation for 24 h. Normal HDF complete medium was used as the control. The extracts were then removed, and 100 μL of a 1× solution of resazurin sodium salt in complete medium was added to the cells. The cells were incubated for 4 h at 37 °C and 5% CO_2_. After incubation, the solutions containing reduced resorufin were transferred to measure fluorescence intensity at 545 nm excitation and 590 nm emission using a SpectraMax i3x Multi-Mode Plate Reader (Molecular Devices, San Jose, CA, USA). Fluorescence intensity was used as an indicator of cell viability [8].

#### 2.7.3. Hemolysis Assay 

Whole human blood from a healthy donor was collected in blood collection tubes containing citrate. Three samples of films were placed in separate 15 mL tubes, each containing 10 mL of sterile PBS 1×. For controls, PBS 1× served as the negative control (CTRL-), and deionized water was used as the positive control (CTRL+). All samples and controls were incubated at 37 °C for 30 min. Afterwards, 200 μL of diluted blood (4 parts citrated blood mixed with 5 parts PBS 1×) was added to each tube, followed by gentle mixing by inversion. Samples were incubated for 1 h at 37 °C, with additional gentle mixing after 30 min. Post incubation, the tubes were centrifuged at 800 g for 5 min, and 100 μL aliquots of the supernatant were transferred to a 96-well plate for absorbance measurement at 540 nm [8]. Hemolysis was calculated as per Equation (12).

(12) $$Hemolysis=\frac{OD\;samples-OD\;CTRL\;Neg}{OD\;CTRL\;Pos-OD\;CRTL\;Neg}\times 100$$

### 2.8. Statistical Analysis

All the obtained results are expressed as the average values of the experimental measurements. Data averages were analyzed with Statistica^®^ software, version 8 by employing the Tukey test with a 5% significance level.

## 3. Results and Discussion

### 3.1. Chitosan Properties

The chitosans’ molecular weight, polydispersity index and deacetylation degree are shown in Table 2. The chitosans are ordered from C1 (standard), followed by chitosans from lower to higher M_W_.

The chitosans’ average molecular weights, M_W_ and M_N_, range from 110 to 341 kg/mol and 63 to 230 kg/mol, respectively. This range covers low (C2 and C3), medium (C1, C4, C5) and high (C6) molecular weights, according to Sigma-Aldrich definitions [35].

Considering the direct proportionality between viscosity and molecular weight, the extent of this range is especially significant for evaluating the influence of molecular weight (M_W_) on the physicochemical properties and antibacterial effectiveness of the films, thereby assessing the potential for interchangeability among suppliers.

Another relevant property is the samples’ polydispersity index, reported as PDI (M_W_/M_N_), which allows us to evaluate the heterogeneity of a sample based on its size. All samples displayed a PDI < 2, with no significant differences among them. Overall, all the tested chitosans were homogeneous and below the PDI threshold of 3, established for pharmaceutical applications [45].

The degrees of deacetylation (DDAs) varied from 82.2% to 94.7%. C1 and C3 had the lowest and highest DDA values, respectively. The deacetylation degree is an important parameter to be considered to obtain high crosslinking degree. In fact, high DDA values indicate that there are more amino groups available for crosslinking, which should have an impact on the physicochemical performance of chitosan films [46,47]. As observed from Table 2, the samples can be categorized into two distinct groups, one exceeding 90% DDA (C2, C3 and C4) and the other hovering around 83% DDA (C1, C5 and C6). Interestingly, the group with higher DDA values exhibits smaller molecular weights (M_W_), which may be due to the deacetylation process that results in the degradation of chitosan chains [48,49].

In addition to molecular characteristics, chitosan quality depends on its purity, which is related to ash, chloride and protein contents [50]. Lower impurity content indicates higher chitosan quality. Moreover, the highly hygroscopic property of chitosan is correlated to the moisture content. The values determined for those parameters are presented in Table 3.

The chitosan samples exhibited moisture content ranging from 7 to 11.7% (around 5% of variation). Typically, commercial chitosan contains less than 10% in moisture [51]; thus, C3 and C5 are higher than the average reported value. Moisture control is important for obtaining reproducible chitosan products. However, implementing a drying process on industrial scale may increase significantly production costs and affect the quality of products [52], which should be avoided.

Chitosan production involves chitin deproteinization and demineralization, typically by adding NaOH, HCl and NaClO to the raw material. The efficiency of the process can be monitored by measuring residuals from this process in the form of proteins, ash and chloride [37]. Residual proteins, which could represent a source of toxicity, were quantified by elemental analysis, and no quantifiable protein was detected according to this method. These results are in accordance with health and pharmaceutical applications that establish a limit for proteins of 0.1–0.2% [45,53]. Ash content varied from 0.6 to 1.5%, without exceeding the maximum 1.5% recommend for biomedical applications [45]. Remarkably, the samples C2, C3 and C6 displayed values below 1%. Finally, chloride content ranged from 0.3 to 1.2%. The maximum recommended value for chitosan-based products is less than 1%, which is set to avoid an inflammatory response [54]. Therefore, the value recorded for chitosan C4 is concerning.

The presence of heavy metals was also assessed (Table 4) due to their potential toxicity effect on humans at high levels, particularly when considering applications in contact with human blood. Heavy metals can be present in chitin due to water contamination in the source habitat. Interestingly, they can be found even after chitosan production [55]. Chitosans were tested for arsenic (As), cadmium (Cd), cobalt (Co), chromium (Cr), copper (Cu), mercury (Hg), nickel (Ni), lead (Pb) and tin (Sn). Among them, Cd, Hg and Pb are considered priority metals to public health, due to their high toxicity [56]. However, their concentrations could not be estimated as they were below the limit of quantification. Detectable levels were observed only for Co, Ni and Cu. Co was only observed in C5 and C6, both below 0.2 ppm, the acceptance criteria for chitosan according to the *United States Pharmacopeia* [45]. Ni content exceeded accepted levels for all samples, over 1 ppm, with values from 6 to 16 ppm, in C1 and C5, respectively [45]. Cu content ranged from 1 to 12 ppm, which is below the limit of 40 ppm according to the *Pharmacopeia* [45,53].

### 3.2. Film Characterization

After assessing the main chitosan characteristics that may impact physicochemical and biological performance of chitosan products, the films were produced and analyzed in terms of thickness, swelling, mass loss and water contact angle. The results are presented in Table 5.

The films’ thicknesses were similar for all samples, varying slightly from 19.8 µm to 22.4 µm, similar to that observed previously by Chevallier et al. [8]. Other studies have also shown that thickness does not depend on the chitosan molecular weight [29,57].

Swelling values were similar for C1 to C5 (104.2% to 126.4%), while C6 presented a value that was twice as high (206.5%). The reason for this difference may be due to the higher molecular weight, with consequent entangled chain conformation that reduces the availability of amino groups for crosslinking. As a result, the chitosan chains in C6 films would exhibit a higher chain relaxation potential and, thus, higher swelling [58,59]. In addition, C6 belongs to the group of lower DDA (Table 2), which may have resulted in a lower degree of crosslinking and, consequently, higher water uptake.

The stability of the films was studied in terms of mass loss in PBS and showed a variation from 21.1% to 29.6% for C5 and C6, respectively. It is known that mass loss can be correlated to both DDA and M_W_. Indeed, as can be seen from Table 2, C6 is the group of lower DDA, which means more acetyl groups (-NHCOCH_3_) that lead to a more amorphous area that degrades faster than the crystalline zone, as reported by Hsu et al. [60]. Moreover, as can be seen from Table 5, the highest M_W_ (C6) was also the highest water uptake, which could accelerate degradation through mass loss. On the other hand, no clear tendency was observed for the other chitosans to explain their mass loss behavior.

The contact angle showed close values for all samples, varying from 95° to 100°, meaning that the differences in the chitosans’ physicochemical properties did not affect the surface wettability of the films.

### 3.3. Antibiotic Release

**Figure 1:**
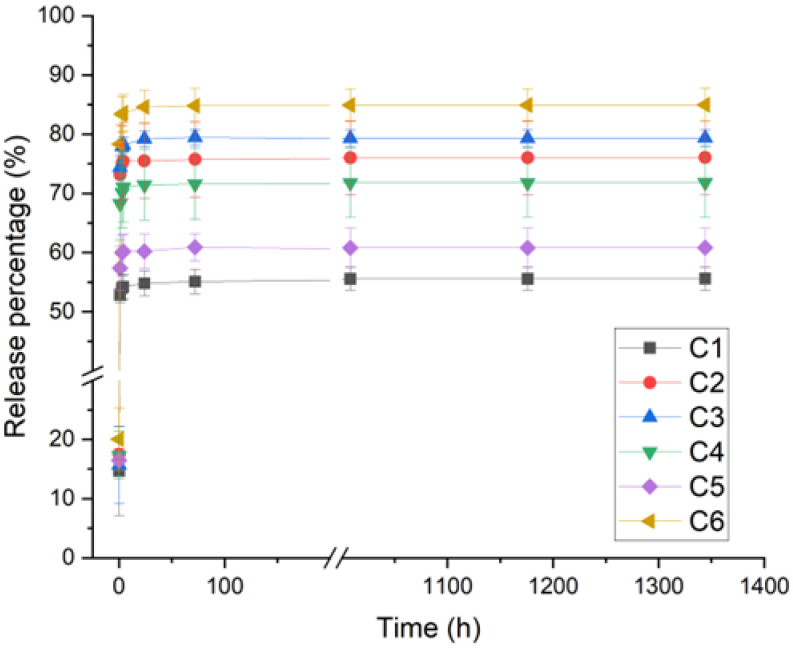
Release kinetics of different chitosan suppliers’ films loaded with gentamicin and crosslinked with tannic acid and iron sulfate as a cumulative percentage of the release over time.

Chitosan films loaded with the antibiotic GS were characterized in terms of release kinetics, and the results are shown in Figure 1.

All the films showed an initial burst release in the first hour; however, it was less pronounced than non-crosslinked films (Figure S1). GS was released at different percentages among the films. Interestingly, C6 chitosan, which shows the highest M_W_, swelling and, therefore, more polymer chain relaxation, also displayed the highest percentage of GS release after 1400 h. The lowest GS release was observed for C1 and C5 which, indeed, showed lower M_W_ and swelling degrees compared to C6. DDA was similar among C1, C5 and C6, which could mean that this parameter does not significantly affect GS release.

In turn, for C2, C3 and C4, which have the highest DDA and the lowest M_W_, it was expected that they also showed less swelling and, consequently, lower GS release. However, this tendency was not observed. Therefore, swelling, M_W_ and DDA parameters seem not to be enough to explain the different observed GS released percentages among all the studied chitosans.

In this sense, a closer look was taken at other parameters, specifically impurities (ash copper (Cu)) and moisture levels. It was observed that C6 is among the purest samples, having low ash, Cu and moisture levels. On the other hand, C1 and C5 exhibiting among the highest ash, Cu and moisture contents. This suggests that the net amount of chitosan for C1 and C5 films contain less chitosan compared to C6. Since the amount of crosslinker is the same for all films, C1 and C5 probably have a higher crosslinking degree and, therefore, a lower GS release percentage. Furthermore, copper (Cu) is known to form a complex with chitosan, which should also increase the crosslink degree, and it is difficult to precisely state which phenomena is more prominent.

C2, C3 and C4 have similar values of GS release percentages, and variable levels of moisture, ash and Cu. Particularly, C3 has the highest moisture level of all the tested chitosans, presenting also high Cu content, but it is the purest in terms of ash content, which is thought to have brought the GS release to an intermediate value.

In general, it was observed that moisture and impurity levels had a significant effect on GS release percentage from the chitosan films. M_W_ was also shown to have some impact, while DDA had almost no impact. Nevertheless, all samples showed similar sustained GS release after the burst. Therefore, changing chitosan supplier affected the burst but did not significantly influence the release kinetics over a prolonged period.

### 3.4. Antibacterial Activity Results

**Figure 2:**
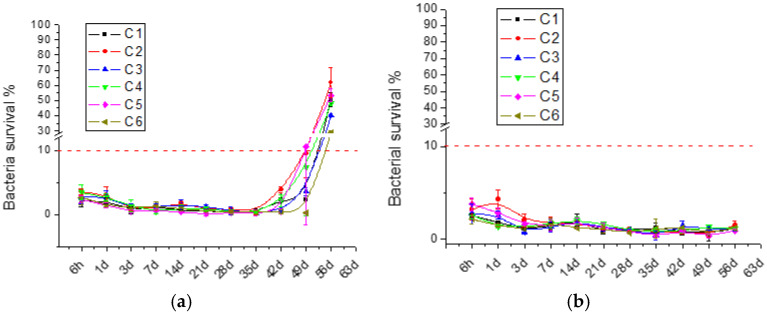
Bacterial survival of (**a**) *E. coli* and (**b**) *S. aureus*, indirect assay performed in in MHB media after incubation with film eluates at different time points. The dotted line represents the contamination threshold of 10%.

The antibacterial activity exerted by the released GS was studied over time. The results are shown in Figure 2.

This assay was adapted from the MIC_90_ evaluation. Therefore, films were considered active when bacteria survival was less than 10% [8]. Positive and negative controls showed 0% and 100% bacteria survival, respectively. Pure chitosan films were analyzed previously [34], and as they surprisingly showed no antibacterial activity, they were not included as controls in this study. This could be explained by the fact that the antibacterial activity of chitosan is attributed to its positive amine charge, which interacts with bacterial membranes or binds to bacterial DNA, both mechanisms resulting in bacterial death. However, during film formation and crosslinking, chitosan’s intrinsic antibacterial activity diminishes as its amino groups are hindered. On the contrary, regardless the chitosan source, GS-loaded films displayed antibacterial activity against *E. coli* for 42 days and against *S. aureus* for more than 56 days, underlying the importance of the gentamicin release. The difference in activity between the Gram-positive *S. aureus* and Gram-negative *E. coli* can be attributed to the strain susceptibility to the antibiotic. These results confirm that, despite the initial substantial burst release showed in Figure 1, the released GS concentrations were above the MIC value (0.25 μg/mL) [61]. Therefore, antibacterial activity over time is similar among all chitosan films. This result confirms the interchangeability between the different tested suppliers, aiming at antibacterial film applications.

### 3.5. Biocompatibility Results

**Figure 3:**
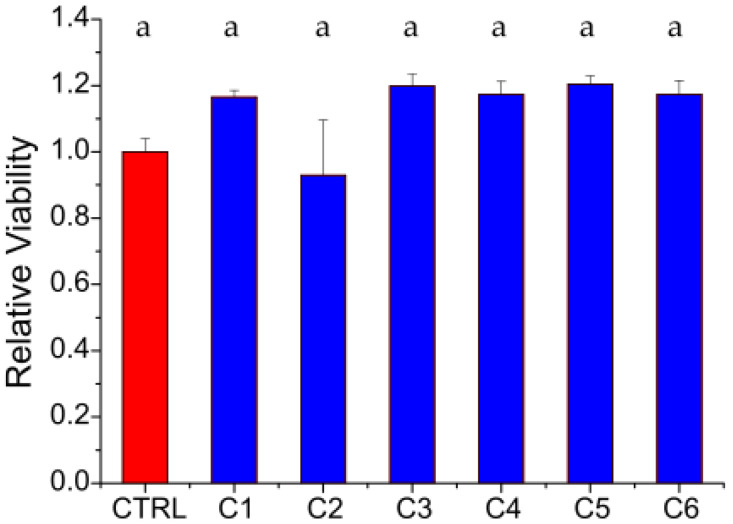
Indirect cytotoxicity assay results obtained by treating HDFs with the extracts from the films. Cell viability was measured after 1 day of incubation by means of a resazurin salt solution assay. The results displayed in blue have been normalized to the control (CTRL) represented in red. The letter “a” indicates that all samples belong to the same group (no significant statistical difference, Tukey test *p* < 0.05).

Finally, the films’ indirect cytotoxicity and hemolytic activity were assessed. Indirect toxicity results are shown in Figure 3.

According to Figure 3, the films’ extracts did not exert any negative effects on the viability of the treated HDFs after 1 day of incubation. Despite the differences observed in both physicochemical properties and impurity contents among the tested chitosans, the cytocompatibility values are similar (*p* < 0.05). These results are in agreement with Abedian et al., who found that chitosan’s molecular weight did not affect cytotoxicity against fibroblasts [62].

**Figure 4:**
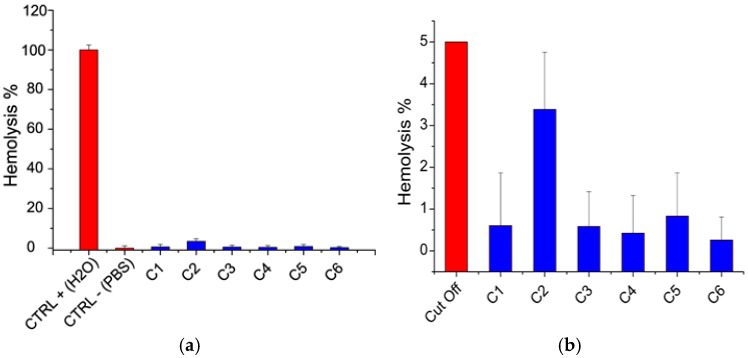
Hemolysis test. (**a**) Results (blue bars) compared to positive and negative controls (CTRL+, CTRL-) (red bars). (**b**) Results (blue bars) compared to the 5% hemolysis cut off (red bar).

Hemocompatibility was assessed based on the hemolysis test. According to ASTM [63], a material is considered hemolytic if it induces hemolysis at percentages higher than 5%. The results of the hemolysis test are shown in Figure 4.

It was noticed that hemolysis percentage was below 5% for all films, indicating that they can be considered hemocompatible (Figure 4).

## 4. Conclusions

In this study, six chitosans from different suppliers were compared in terms of their physicochemical properties and used to produce antibacterial films loaded with gentamicin. The chitosans had average molecular weights (M_W_) from 110 to 341 g/mol, ranging from low to high M_W_. The degree of deacetylation (DDA) ranged from 82.2 to 94.7%. Gentamicin release showed an initial burst, which varied among the samples, followed by similar gradual and sustained release for all films. Both M_W_ and DDA seemed to have much less impact on bursting compared to moisture and impurities contents, especially ash and copper. Antibacterial activity over time was similar for all samples, being observed up to 42 days against *E. coli* and for more than 56 days against *S. aureus*. Moreover, the films were shown to be cyto- and hemocompatible.

In general, the results obtained in this study showed that chitosan feedstock selection does not significantly impact the obtained films in terms of their antibacterial efficacy, biocompatibility and overall release profile. However, to fine-tune the antibiotic burst release, it is recommended to measure chitosan’s impurities, especially ash and copper, which were found to have the highest impact. Therefore, considering chitosan-based antibacterial films production, the interchangeability among the studied suppliers is feasible. This study is motivating in terms of the translation from bench to industrial production of these chitosan-based films.

## Figures and Tables

**Table 1:** Chitosan suppliers, acronyms and characteristics given by the suppliers.

Supplier	Chitosan	Viscosity (cP)	DDA (%)
Sigma	C1	331	≥75%
Chitolytic	C2	75	≥90%
Zhejiang	C3	36	≥95%
Fingres	C4	138	≥95%
Quitomax	C5	308	≥75%
Jiangsu	C6	1100	≥75%

**Table 2:** Weight average molecular weight (M_W_), number average molecular weight (M_N_), polydispersity index (PDI) and DDA of chitosan from different suppliers.

Chitosan	M_W_ (kg/mol)	M_N_ (kg/mol)	PDI	DDA (%)
C1 *	217 ± 7 ^b^	165 ± 4 ^b^	1.3 ± 0.1 ^a^	82.2 ± 1.2 ^a^
C2	110 ± 2 ^a^	63 ± 4 ^a^	1.8 ± 0.1 ^a^	91.9 ± 2.1 ^b^
C3	114 ± 3 ^a^	76 ± 3 ^a^	1.6 ± 0.1 ^a^	94.7 ± 0.1 ^b^
C4	193 ± 7 ^b^	138 ± 17 ^b^	1.4 ± 0.1 ^a^	90.6 ± 2.0 ^b^
C5	201 ± 3 ^b^	113 ± 18 ^ab^	1.8 ± 0.3 ^a^	83.5 ± 1.2 ^a^
C6	341 ± 17 ^c^	230 ± 13 ^c^	1.5 ± 0.1 ^a^	83.7 ± 2.9 ^a^

* C1—reference chitosan from Sigma. Different letters in the same column indicate significant difference (Tukey test *p* < 0.05).

**Table 3:** Impurity content in chitosan samples.

Chitosan	Moisture (%)	Ash (%)	Cl^−^ (%)
C1	9.14 ± 0.15 ^a^	1.37 ± 0.03 ^a^	0.88 ± 0.18 ^ab^
C2	7.04 ± 0.19 ^c^	0.89 ± 0.15 ^c^	0.35 ± 0.18 ^c^
C3	11.69 ± 0.48 ^b^	0.63 ± 0.06 ^b^	1.00 ± 0.10 ^ab^
C4	8.99 ± 0.22 ^a^	1.34 ± 0.16 ^a^	1.18 ± 0.10 ^b^
C5	11.59 ± 0.21 ^b^	1.49 ± 0.02 ^a^	0.77 ± 0.10 ^a^
C6	8.81 ± 0.18 ^a^	0.78 ± 0.04 ^bc^	0.30 ± 0.10 ^c^

Different letters (^abc^) in the same column indicate groups with significant difference (Tukey test *p* < 0.05).

**Table 4:** Heavy metals content in chitosan samples, the amounts are in µg/g.

Chitosan	As	Cd	Co	Cr	Cu	Hg	Ni	Pb	Sn
C1	nd	nd	nd	nd	5.0 ± 2.6	nd	5.9 ± 0.9	nd	nd
C2	nd	nd	nd	nd	1.1 ± 0.2	nd	13.0 ± 3.0	nd	nd
C3	nd	nd	nd	nd	5.3 ± 0.9	nd	8.7 ± 1.3	nd	nd
C4	nd	nd	nd	nd	2.8 ± 0.1	nd	14.3 ± 0.2	nd	nd
C5	nd	nd	0.1 ± 0.1	nd	11.7 ± 0.1	nd	16.0 ± 0.2	nd	nd
C6	nd	nd	0.1 ± 0.1	nd	1.3 ± 0.1	nd	8.2 ± 1.1	nd	nd

nd—not detected. The limit of quantification for each metal was as follows: arsenic (As) = 0.01 µg/g, cadmium (Cd) = 0.001 µg/g, cobalt (Co) = 0.001 µg/g, chromium (Cr) = 0.05 µg/g, copper (Cu) = 0.001 µg/g, mercury (Hg) = 0.01 µg/g, nickel (Ni) = 0.01 µg/g, lead (Pb) = 0.02 µg/g and tin (Sn) = 0.06 µg/g. Note: 1 µg/g = 1 ppm.

**Table 5:** Thickness, swelling, mass loss and contact angle of the films.

Chitosan	Thickness (µm)	Swelling (%)	Mass Loss (%)	Contact Angle (°)
C1	19.8 ± 2.2 ^a^	126 ± 14 ^a^	26.7 ± 0.7 ^ab^	96 ± 2 ^a^
C2	21.4 ± 2.4 ^a^	121 ± 10 ^a^	28.7 ± 1.6 ^b^	95 ± 3 ^a^
C3	22.2 ± 1.5 ^a^	104 ± 11 ^a^	23.7 ± 3.0 ^ab^	96 ± 2 ^a^
C4	22.0 ± 2.0 ^a^	123 ± 3 ^a^	21.9 ± 2.9 ^a^	100 ± 1 ^a^
C5	20.8 ± 1.8 ^a^	104 ± 11 ^a^	21.1 ± 3.1 ^a^	97 ± 3 ^a^
C6	22.4 ± 1.1 ^a^	206 ± 28 ^b^	29.6 ± 1.6 ^b^	96 ± 1 ^a^

Different letters (^ab^) in the same column indicate groups with significant difference (Tukey test *p* < 0.05).

## Data Availability

Data available in a publicly accessible repository https://drive.google.com/drive/folders/1m9t-Ah_S30uMogDtp4Y5VBkivWDiiyZ_?usp=sharing (accessed on 25 March 2025).

## Supplementary Materials

The following supporting information can be downloaded at: https://www.mdpi.com/article/10.3390/polym17070884/s1, Figure S1: Gentamicin release from films made of different chitosans without crosslinking.
