# Supplementary material for: Sourcing Interchangeability in Commercial Chitosan: Focus on the Physical–Chemical Properties of Six Different Products and Their Impact on the Release of Antibacterial Agents
Source: Polymers (Basel). 2025 Mar 26;17(7):884. doi: 10.3390/polym17070884 (PMC11991257; doi:10.3390/polym17070884)
Supplement: Supplementary file 1 [file polymers-17-00884-s001.zip › polymers-3515941-supplementary.pdf]

polymers

MDPI

*Article*

# Sourcing interchangeability in commercial chitosan: Focus on physical-chemical properties of six different products, and their impact on the release of antibacterial agents

**Isabela T. Rampim[1], Helton José Wiggers[1*], Cecilia Z. Bueno[1], Pascale Chevallier[2], Francesco Copes[2] and Diego Mantovani[1,2*]**

[1] Laboratory for Biomaterials and Bioengineering (LBB-BPK), Associação de Ensino, Pesquisa e Extensão BIOPARK, Max Planck Avenue, 3797, Building Charles Darwin, Toledo 85919-899, PR, Brazil
[2] Laboratory for Biomaterials and Bioengineering (LBB-UL), Canada Research Chair Tier I, Department of Min-Met-Materials Engineering & CHU de Quebec Research Center, Division Regenerative Medicine, Laval University, Quebec City, QC G1V0A6, Canada
* Correspondence: diego.mantovani@gmn.ulaval.ca, helton.wiggers@bpkedu.com.br

**Abstract:** Sourcing and batch-differences are often cited as intrinsic drawbacks for all natural polymers. Chitosan makes no exception. Chitosan is a biocompatible and biodegradable biopolymer with high potential for several biomedical applications, and especially for releasing drugs, bactericidal and virucidal agents. Despite the potential of chitosan as a matrix for producing antibacterial films, the variability in its composition, stemming from its natural sources, can hinder the translation from bench to industry. To overcome this concern, we conducted a study to access the interchangeability of chitosan for the development of antibacterial drug release systems, in particular one system crosslinked with tannic acid and iron sulfate. Chitosans from different suppliers were characterized and used to synthetize films containing gentamicin, according to a previously reported protocol. The impact of molecular weight (MW), deacetylation degree and purity on film properties and antibiotic release kinetics was assessed and results were compared. Chitosan molecular weight varied from 110 to 340 g/mol, degree of deacetylation from 82.2 to 94.7%, moisture from 7.04 to 11.69 %, ash from 0.63 to 1.49 % and Cu from 1.1 to 11.7 μg/g. Protein content was not quantifiable in any of the samples. The obtained films had thickness ranging from 19.8 to 22.4 μm, mass loss in PBS from 21.1 to 29.6 % and swelling in PBS from 104.2 to 206.5 %. The films exhibited different initial burst followed by similar sustained release profiles. All films exhibited antibacterial activity against both E. coli and S. aureus for at least 42 days. Moreover, films were cyto- and hemocompatible. Therefore, despite some differences in physicochemical properties, the interchangeability among the studied chitosan suppliers to produce antibacterial films is feasible, and the final product properties and performances are not significantly altered.

**Keywords:** Natural polymers; Chitosan; Interchangeability; Properties

Additional information to

### 3.3. Antibiotic release

The release kinetics study was performed similarly to the procedure described in the main text, up to 3 days. The films characterized herein were produced without crosslinker by mixing, under magnetic stirring, 6.67 mL of chitosan solution at 15 mg/mL in acetic acid 1% v/v, 1.0 mL of gentamicin sulphate at 10 mg/ml and ultrapure water up to a final volume of 10 mL. The samples were poured into 90 mm petri dishes and dried at 37ºC until constant mass. Figure 1S shows the cumulative gentamicin release percentage against time.

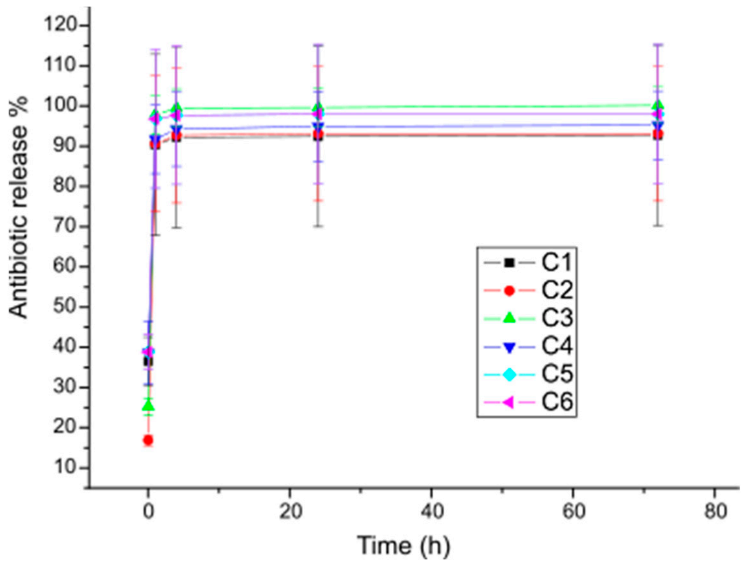


Figure S1. Gentamicin release from films made of different chitosans without crosslinking.

Gentamicin is released at similar rates from films without crosslinking, therefore is reasonable assume that gentamicin has no interaction with chitosan, and the crosslinking is essential to control the antibiotic release.

**Disclaimer/Publisher's Note:** The statements, opinions and data contained in all publications are solely those of the individual author(s) and contributor(s) and not of MDPI and/or the editor(s). MDPI and/or the editor(s) disclaim responsibility for any injury to people or property resulting from any ideas, methods, instructions or products referred to in the content.
